# Acid-Modified Biochar Derived from Agricultural Waste for Efficiently Capturing Low-Concentration Nitrous Oxide (N_2_O): Mechanisms and Environmental Implications

**DOI:** 10.3390/toxics13080623

**Published:** 2025-07-25

**Authors:** Mingming Fu, Yingdi Ma, Fengrui Yang, Ziyu Xiao, Mei Wang, Shaoyuan Bai, Qin Zhang, Huili Liu, Dandan Xu, Yanan Zhang

**Affiliations:** 1 College of Environmental Science and Engineering, Guilin University of Technology, Guilin 541004, China; mmfu@glut.edu.cn (M.F.); 1020220419@glut.edu.cn (Y.M.); 13149605387@163.com (F.Y.); xiaoziyu0606@163.com (Z.X.); baisy@glut.edu.cn (S.B.); 2003070@glut.edu.cn (Q.Z.); liuhuili@glut.edu.cn (H.L.); dandanxu@glut.edu.cn (D.X.); zyanan@glut.edu.cn (Y.Z.); 2 Guangxi Key Laboratory of Environmental Pollution Control Theory and Technology, Guilin University of Technology, Guilin 541006, China; 3 Hengsheng Water Environment Treatment Co., Ltd., Guilin 541100, China; 4 Guangxi Engineering Research Center of Comprehensive Treatment for Agricultural Non-Point Source Pollution, Guilin University of Technology, Guilin 541006, China; 5 Modern Industry College of Ecology and Environmental Protection, Guilin University of Technology, Guilin 541006, China

**Keywords:** low-concentration N_2_O, biochar, acid modification, adsorption

## Abstract

Low-concentration N_2_O (≤5%) emissions from agricultural fields and waste treatment facilities in China reach 7.333 × 10^5^ t annually, making them a significant but inadequately controlled contributor to global warming. Agricultural wastes were selected as precursors to prepare biochar, including pecan shell (SH), poplar sawdust (JM), wheat straw (XM), and corn straw (YM), which were subsequently acid-modified with 0.1 mol L^−1^ HCl. The objectives were (i) to quantify the enhancement in N_2_O capture achievable by acid treatment, (ii) to elucidate the underlying chemisorption mechanism, and (iii) to identify the most efficient feedstock for practical deployment. Acid modification increased the oxygen content, specific surface area, and the number of hydroxyl and carboxyl groups on the biochar surface. Both modified and unmodified biochar followed the pseudo-second-order kinetic model (R^2^ ≥ 0.960), indicating chemisorption-dominated processes. The adsorption performance ranked as XM > JM > SH > YM, with XM exhibiting the highest adsorption capacity (26.000 mol/kg unmodified, 43.088 mol/kg modified, 65.72% increase). The Langmuir model provided a better fit for N_2_O adsorption, suggesting dynamic multilayer heterogeneous adsorption. The findings demonstrate that acid-modified biochar derived from agricultural waste is a scalable, economical, and environmentally friendly adsorbent for mitigating low-concentration N_2_O emissions.

## 1. Introduction

As global warming garners increasing attention, reducing greenhouse gas emissions and enhancing their sequestration have become research priorities. Under the United Nations Framework Convention on Climate Change, signatory nations have incorporated methane (CH_4_), nitrous oxide (N_2_O), hydrofluorocarbons (HFCs), perfluorocarbons (PFCs), sulfur hexafluoride (SF_6_), and nitrogen trifluoride (NF_3_) (collectively termed “non-CO_2_ greenhouse gases”) into emission reduction frameworks [1]. Although N_2_O emissions are relatively low compared to CO_2_ and CH_4_, its warming potential is 298 times greater than CO_2_, contributing approximately 6% to global greenhouse effects [2,3]. Due to technical and cost limitations, low-concentration N_2_O (≤5%) [4,5,6] is often released into the atmosphere without treatment. N_2_O originates from natural processes (57%, e.g., microbial activity in oceans and soils) and anthropogenic sources (43%, e.g., nitrogen fertilizer use, fossil fuel combustion, waste treatment, and biomass burning) [7]. The widespread application of chemical nitrogen fertilizers to croplands has led to long-term increases in agricultural nitrogen emissions [8]. This N_2_O not only damages the ozone layer but also reacts with atmospheric H_2_O and SO_2_ to form acid rain [9], posing severe ecological and health risks. Mitigating anthropogenic N_2_O emissions is among the fastest and most cost-effective strategies to curb its impact.

The application of urea and livestock manure as well as water management in paddy fields are the key processes for nitrous oxide (N_2_O) emissions. Although their emission sources are low (≤5%), the total emissions are significant. Biowheel technology has been confirmed to play a continuous reduction role in these scenarios. By coupling the biowheel with activated sludge or biofilm biochar, it is possible to utilize a low-oxygen zone for simultaneous nitrification–denitrification, which can reduce the gas partial pressure at N_2_O production sites by 30–50% [10]. Biochar, a carbon-rich solid produced from biomass pyrolysis under oxygen-limited conditions [11], exhibits excellent gas adsorption due to its porous structure, high surface area, mechanical stability, and regenerability [12]. However, most raw biochars exhibit relatively weak adsorption capacity, requiring modification to enhance their adsorption ability [13]. Studies have confirmed that acid modification (e.g., HCl treatment) enhances biochar’s surface properties by reducing ash content, thereby improving N_2_O adsorption [14]. For instance, some scholars have pointed out that the adsorption of low-concentration nitrate nitrogen by modified aquatic plant biochar is significantly higher [15]. Meanwhile, functional groups such as carboxyl groups (-COOH), phenolic groups, and hydroxyl groups (-OH) on the surface of biochar can undergo chemical reactions with N_2_O molecules, thereby enhancing the adsorption performance [16]. Another study indicates that acid modification treatment can increase the specific surface area and pore volume of biochar by four to six times compared to the original material, while promoting the formation of polar-oxygen-containing functional groups on the surface [17]. These findings provide valuable insights, but there are relatively few studies on the direct adsorption performance of biochar and acid-modified biochar for low-concentration N_2_O gas, despite their huge potential [18].

This study employed four types of agricultural waste as precursors to prepare biochar via high-temperature anaerobic pyrolysis, including pecan shell, poplar sawdust, wheat straw, and corn straw. The biochar’s adsorption performance was enhanced through combined acid–alkali and acid modification methods. We characterized the physicochemical properties of the biochar, both before and after modification using elemental analysis, including X-ray diffraction (XRD), Fourier-transform infrared spectroscopy (FTIR), Brunner−Emmet−Teller (BET), and scanning electron microscopy (SEM). Kinetic and isothermal adsorption experiments were conducted to evaluate the effects of acid modification on the rate and capacity of biochar for N_2_O adsorption at varying concentrations. Furthermore, changes in N_2_O gas adsorption were monitored to elucidate the intrinsic mechanisms governing the adsorption performance of pristine and modified biochar. This study aims to identify an effective biochar material for improving N_2_O gas adsorption capacity.

## 2. Materials and Methods

### 2.1. Preparation of Biochar and Gas Source

Four kinds of agricultural wastes with different structural characteristics, such as pecan shell, poplar sawdust, wheat straw, and corn straw, were selected as raw materials for preparation. To minimize environmental microbial interference with the adsorption of low-concentration N_2_O gas, the raw materials were sterilized in a 121 °C autoclave for 30 min over two consecutive days. After drying and crushing, the materials were sieved through a 100-mesh screen and prepared using a tubular furnace. Nitrogen (N_2_) was used as the protective gas, and appropriate firing temperatures, heating rates, and holding times were determined through orthogonal experiments. The biochar preparation temperature, heating rate, and holding time are shown in Table 1. The prepared biochar was repeatedly rinsed with deionized water until the pH of the rinse water matched that of the deionized water. The washed biochar was then placed in a blower drying oven and dried at 105 °C for 24 h before being stored for use. For the acidic-modified biochar, 0.1 mol/L HCl solution was used as the modification solution. The biochar was placed in a beaker with a ratio of 1:50 between the biochar and the solution. The mixture was stirred at 200 r/min for 24 h in a constant-temperature shaker set at 60 °C. After filtration, the solid samples were washed with deionized water until the wash solution reached a pH of about 7; after acid washing, the solids were dried at 105 °C for 24 h and stored for further use, resulting in acid-modified biochar labeled as SH-SG, JM-SG, XM-GM, and YM-SG. The low-concentration N_2_O gas (100.5 ppm, 99.999% purity) used in the experiments was supplied by Hongrun Technology Equipment Co., Ltd. (Guilin, China).

### 2.2. Characterization of Biochar

Precisely 5.000 mg of dried biochar was weighed using a microbalance and placed in a preheated elemental analyzer (1150 °C) that had undergone rigorous leak testing. The contents of carbon (C), nitrogen (N), and hydrogen (H) were determined, while the oxygen (O) content was measured separately using the O mode. An appropriate amount of dried sample was placed in the sample cell and its phase was analyzed using an X-ray diffractometer with a scanning angle (2θ) of 5–90°. The analysis was performed using Jade 6.5 software. The surface functional groups of biochar materials were analyzed and determined by Fourier-transform infrared absorption spectrometer. The specific surface area and pore characteristics were determined using gas adsorption analysis (America Micromeritics ASAP 2460, N_2_ 77 K). Approximately 200 mg of dried sample was placed in a glass sample tube, pretreated under vacuum with heating at 200 °C for 8 h, and subjected to pore analysis. Afterward, the cold free-space coefficient was measured. Following the test, the tube was pressurized with N_2_ to 77 kPa and reweighed, and the obtained Q-value average and mass were input into the micropore analysis workstation for data processing. The dry samples were adhered to the conductive adhesive in a single layer by laying it flat, and then the samples were wrapped with platinum film to enhance their conductivity. After the sample was prepared, the surface morphology of the biochar material was observed with a scanning electron microscope.

### 2.3. Kinetic Adsorption Analysis

A 300 mL serum bottle equipped with a butyl rubber stopper was selected as the reaction vessel. This flexible container can adjust its volume according to the internal gas pressure. Precisely 5.00 g of modified biochar was weighed using an analytical balance and placed into the serum bottle. The bottle was then sealed with a rubber stopper and wrapped with PTFE tape to ensure gas-tight conditions. To achieve N_2_O concentrations of 1%, 3%, and 5% (*v*/*v*), 3 mL, 9 mL, and 15 mL of ambient air were extracted from the bottle using a syringe and replaced with an equivalent volume of pure N_2_O (99.999% purity). All experiments were conducted at room temperature. Each treatment was performed in triplicate, with a blank control (containing no biochar) included for N_2_O concentration calibration. The reaction was initiated upon gas injection. Gas samples (3 mL) were collected at 0, 3, 6, 10, 20, 30, 60, and 120 min using a 5 mL syringe and transferred into gas sampling bags for subsequent analysis. The N_2_O concentration in the collected samples was quantified using a GC-7890B gas chromatograph by Agilent Technologies (China) Co., Ltd. The adsorption kinetics were fitted using both the pseudo-first-order (PFO) equation and the pseudo-second-order (PSO) equation. The pseudo-first-order equation is

(1) $$q_{t}=q_{e}(1-e^{{-k}_{1}t})$$

The pseudo-second-order equation is

(2) $$q_{t}=\frac{q_{e}^{2}k_{2}t}{1+k_{2}q_{e}t}$$

$q_{t}$—amount adsorbed at time *t*, mol/kg;

$q_{e}$—equilibrium adsorption capacity, mol/kg;

$k_{1}$—pseudo-first-order adsorption rate constant, L/min;

$k_{2}$—pseudo-second-order adsorption rate constant, kg/mol/min.

### 2.4. Isothermal Adsorption Analysis

Precisely 5.00 g of the material was weighed using an analytical balance and placed into a 300 mL serum bottle. The headspace N_2_O concentrations were set at 1%, 2%, 3%, 4%, 5%, and 6% (*v*/*v*). Each bottle was sealed with a rubber stopper and wrapped with sealing tape to prevent gas leakage. A blank control was included for each concentration to verify initial N_2_O levels. All treatments were conducted in triplicate with three independent replicates, and the average values were used for analysis. The samples were incubated in a constant-temperature shaker at 30 °C and 160 rpm for 60 min. Subsequently, 3 mL of gas was extracted from each bottle using a 5 mL syringe and stored in gas sampling bags. The N_2_O concentrations were analyzed using a GC-7890B gas chromatograph. The Langmuir and Freundlich isotherm adsorption models were used to analyze the adsorption data. The Langmuir isotherm model, which assumes that the adsorption system is in dynamic equilibrium and the material surface is uniform, effectively describes the adsorption curve. In contrast, the Freundlich isotherm model, an empirical equation, does not make such assumptions and accounts for the non-uniformity of the adsorbent surface [23].

The Langmuir isotherm equation is

(3) $$q_{e}=\frac{q_{m}k_{l}c_{e}}{1+k_{l}c_{e}}$$

The Freundlich isotherm equation is

(4) $$q_{e}=k_{F\;}{p\;}_{e}^{\frac{1}{n}}$$

$p_{e}$—equilibrium pressure, kpa;

$q_{e}$—equilibrium adsorption capacity, mol/kg;

$q_{m}$—monolayer saturation capacity, mol/kg;

$k_{l}$—Langmuir adsorption equilibrium constant, L/kPa;

$k_{F}\backslash n$—Freundlich adsorption equilibrium constant and linearity index.

## 3. Results and Discussion

### 3.1. Characterization and Discussion of Biochar and Acid-Modified Materials

#### 3.1.1. Elemental Analysis and Discussion of Biochar and Acid-Modified Materials

Table 2 shows the elemental compositions of unmodified SH, JM, XM, and YM. SH exhibited the highest carbon content at 85.72%, followed by JM (68.04%), YM (42.92%), and XM (37.84%). JM exhibited the highest oxygen content among these materials at 12.34%, followed by SH (8.85%), YM (7.38%), and XM (6.86%). Hydrogen content was low in all materials, with the highest value of 2.87% in SH. Nitrogen content was also relatively low, with YM having the highest value at 3.19%. Additionally, the H/C, O/C, and (O + N)/C ratios of these materials vary, reflecting differences in their physicochemical structures. Lower H/C ratio indicates higher aromaticity [24], while elevated O/C and (O + N)/C ratios suggest greater oxygen-containing functional groups and higher surface hydrophilicity [25]. Among the four materials, YM has the highest aromaticity and the most oxygen-containing functional groups, leading to higher hydrophilicity, whereas XM shows the lowest aromaticity, and SH exhibits the fewest oxygen-containing functional groups and higher hydrophobicity.

Table 3 depicts the elemental compositions of the materials post-acid-modification. For SH-SG, the carbon content decreased marginally to 83.11%, while the oxygen content increased significantly to 10.91%; hydrogen and nitrogen contents dropped to 2.47% and 0.30%, respectively. JM-SG showed an increase in carbon content to 81.65%, a slight reduction in oxygen to 10.88%, and decreases in hydrogen (1.84%) and nitrogen (0.23%). XM-SG and YM-SG exhibited modest carbon content increases (38.17% and 44.87%, respectively), with oxygen contents rising to 8.17% and 7.99%, hydrogen to 1.84% and 1.29%, and nitrogen decreasing to 0.87% and 0.66%. Post-modification, JM-SG displayed the highest aromaticity, whereas XM-SG had the lowest. SH-SG, XM-SG, and YM-SG showed enhanced hydrophilicity, while JM-SG—despite a slight oxygen content reduction—remained second in oxygen content among the materials. These results indicate that acid modification significantly affects biochar oxygen content, with a comparatively minor impact on carbon content. The decrease in carbon content and the increase in oxygen content may result from HCl treatment, where H^+^ ions oxidize unsaturated carbon on biochar surfaces. This reaction enhances oxygen-containing functional groups (e.g., carboxyl, hydroxyl, lactone), raising the oxygen proportion. Concurrently, decarbonization gases like CO_2_ and CO are released, leading to a relative decline in carbon content [26].

Collectively, the above analyses reveal significant discrepancies in the elemental compositions of the materials before modification, primarily attributed to their inherent chemical structures. The modified biochars exhibit elevated oxygen contents and reduced carbon contents, which may enhance their performance in applications such as adsorption and catalysis.

#### 3.1.2. XRD Analysis and Discussion of Biochars and Acid-Modified Materials

**Figure 1:**
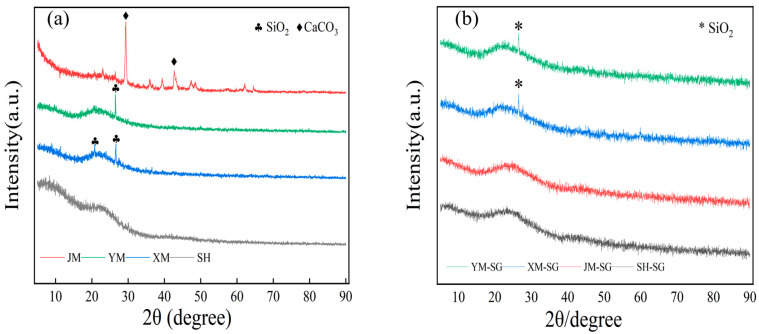
XRD images of biochar before and after modification. (**a**) Before modification, (**b**) after modification.

Figure 1a shows the XRD patterns of biochars before modification. Except for SH, all samples showed distinct peaks within the 2θ range of 22° to 43°. YM exhibited a silicon dioxide (SiO_2_) peak at 26°, while XM had SiO_2_ peaks at 22° and 26°. These peaks are associated with graphitization in carbon materials. JM showed calcium carbonate (CaCO_3_) peaks at 29° and 43°, indicating residual carbonates. JM’s higher peak intensity suggests greater crystallinity and graphitization. Overall, pre-modification crystal structures varied significantly among biochars, likely due to feedstock composition and pyrolysis conditions.

Figure 1b depicts the XRD patterns of the acid-modified biochars. A notable attenuation of the SiO_2_ diffraction peaks within the 2θ range of 22°~26° was observed, indicating a further reduction in graphitization degree induced by acid treatment. The complete disappearance of CaCO_3_’s characteristic peaks in JM-SG post-modification can be ascribed to the exhaustive dissolution or transformation of carbonate minerals during acid washing [27]. These findings collectively underscore the pronounced impact of acid modification on the biochars’ crystalline structures and mineral compositions, which is plausibly mediated through the introduction of oxygenated functional groups and the alteration of mineral phases.

The preceding analyses collectively reveal significant discrepancies in the pre-modification crystalline architectures of biochars derived from various feedstocks. These differences can primarily be attributed to variations in the chemical composition of the raw materials and pyrolysis parameters. Acid modification substantially remodels the biochars’ crystalline frameworks, reducing graphitization and fostering a more porous surface topography with enhanced pore structural development.

#### 3.1.3. FTIR Analysis and Discussion of Biochars and Acid-Modified Materials

**Figure 2:**
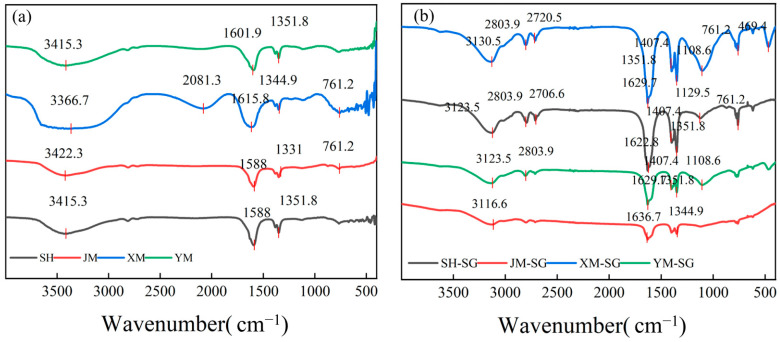
Infrared spectra of biochar before and after modification. (**a**) Before modification, (**b**) After modification.

Figure 2a depicts the FT-IR spectra of biochars before modification. The FT-IR spectra of SH, JM, XM, and YM reveal a broad peak at approximately 3400 cm^−1^, typically associated with O-H stretching vibrations, indicating the presence of -OH functional groups on the biochar surface. These -OH groups may originate from moisture on the biochar surface or result from the oxidation of biochar during pyrolysis. The peak at around 2900 cm^−1^ is assigned to C-H stretching vibrations of alkyl chains, while peaks near 1600 cm^−1^ and 1500 cm^−1^ are attributed to C=C stretching vibrations of aromatic rings, serving as indicators of graphitization degree. Additionally, multiple peaks in the 1000~1300 cm^−1^ region, characteristic of C-O stretching vibrations, suggest the presence of functional groups such as carboxyls (-COOH), alcohols, or ethers [28].

Figure 2b presents the post-acid-modification FT-IR spectra of the biochars. A marked intensification of the O-H stretching vibration band at 3400 cm^−1^ indicates a substantial increase in surface -OH groups following acid treatment. Conversely, the C-H stretching peak at 2900 cm^−1^ exhibited a further decline in intensity, attributable to oxidative scission of alkyl chains during modification. A concomitant reduction in the C=C stretching vibrations of aromatic rings near 1600 cm^−1^ and 1500 cm^−1^ suggests suppression of graphitic ordering. Additionally, a notable enhancement in C-O stretching vibrations in the 1000~1300 cm^−1^ region confirms the introduction of -COOH, alcoholic, or ethereal functional groups [28]. The polarity hierarchy of these moieties (-COOH > -OH > -CH_2_- > -CH_3_) indicates that acid modification elevates surface polarity, thereby enhancing the adsorption affinity for polar N_2_O molecules [29].

#### 3.1.4. BET Analysis and Discussion of Biochars and Acid-Modified Materials

Table 4 presents the BET analysis results of unmodified biochars from four feedstocks. Studies have shown a positive correlation between microporous surface area and adsorption capacity [30]. The specific surface area of SH was 3.3833 m^2^/g, with total pore volume of 0.006687 cm^3^/g, micropore volume of 0.000489 cm^3^/g, and average pore size of 32.0450 nm. JM exhibited the highest specific surface area (190.3343 m^2^/g), total pore volume (0.103057 cm^3^/g), and micropore volume (0.059245 cm^3^/g), with a pore size of 4.6607 nm. XM and YM showed moderate pore structures with specific surface areas of 14.1885 m^2^/g and 134.3059 m^2^/g, respectively. The order of specific surface areas before modification was JM > YM > XM > SH. These findings demonstrate substantial variations in pore characteristics among biochars from different sources, which may be attributed to differences in feedstock chemistry and pyrolysis conditions.

Table 5 presents the BET analysis results of the acid-modified biochars. SH-SG exhibited a remarkable increase in specific surface area to 472.4921 m^2^/g, total pore volume to 0.196466 cm^3^/g, and micropore volume to 0.160959 cm^3^/g, with a concomitant decrease in pore diameter to 3.2040 nm. Similarly, JM-SG showed significant enhancements in specific surface area (312.4882 m^2^/g), total pore volume (0.157832 cm^3^/g), and micropore volume (0.100254 cm^3^/g), accompanied by a reduction in pore diameter to 3.9573 nm. XM-SG and YM-SG also demonstrated notable increases in specific surface area (84.3999 m^2^/g and 203.2276 m^2^/g, respectively), indicating substantial alterations in pore structure post-acid-modification. These results collectively suggest that acid treatment effectively amplifies the specific surface area and micropore volume of biochars, plausibly by promoting graphitization and optimizing pore architecture during the modification process.

Significant differences in the pre-modification pore characteristics of biochars from different feedstocks are primarily associated with the chemical compositions of raw materials and pyrolysis processes. Acid modification notably alters the pore structures of biochars, with more pronounced effects on enhancing specific surface area and micropore volume. The modified biochars exhibit higher specific surface areas and optimized pore architectures, which may endow them with superior performance in applications such as adsorption and catalysis.

#### 3.1.5. SEM Analysis and Discussion of Biochars and Acid-Modified Materials

**Figure 3:**
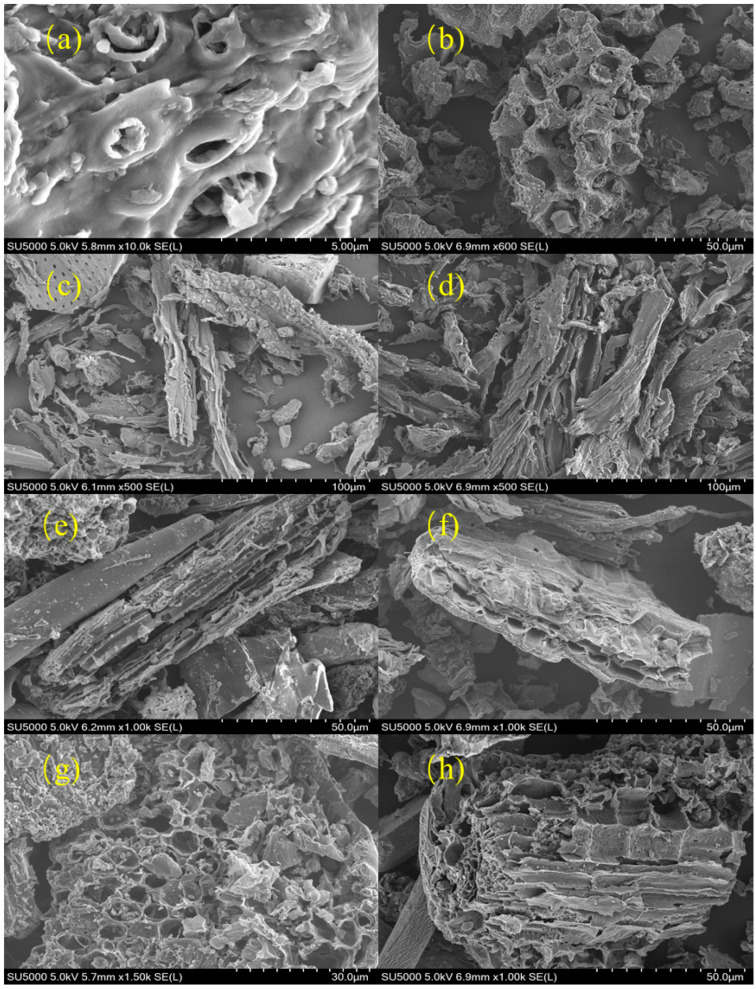
Electron microscope images before and after modification. (**a**) SH, (**b**) SH-SG, (**c**) JM, (**d**) JM-SG, (**e**) XM, (**f**) XM-SG, (**g**) YM, (**h**) YM-SG.

Figure 3 illustrates that pre-modified materials predominantly exhibit rugged, porous surfaces with loosely arranged pore structures characterized by irregular sizes, non-uniform distributions, and potentially poor inter-pore connectivity, which may impede adsorption kinetics. Following acid modification, the materials display more homogeneous and dense surfaces, accompanied by a complex pore network composed of sheet-like pores. The presence of abundant micropores and mesopores further corroborates that acid treatment enhances specific surface area and may improve adsorption selectivity, as pores of diverse dimensions enable the capture of molecules with different sizes. These observations are in agreement with the findings reported by Ke [31].

The pore structures of these materials exhibit diversity and complexity, with distinct surface morphologies and pore size distributions exerting significant impacts on their application performances. Larger pores facilitate higher transport rates, while smaller pores enhance specific surface area and adsorption capacity. Additionally, inter-pore connectivity represents a critical factor: good connectivity improves adsorption kinetics, whereas closed pores may restrict the efficacy of practical application.

### 3.2. Kinetic Analysis and Discussion of Biochars Before and After Acid Modification

**Figure 4:**
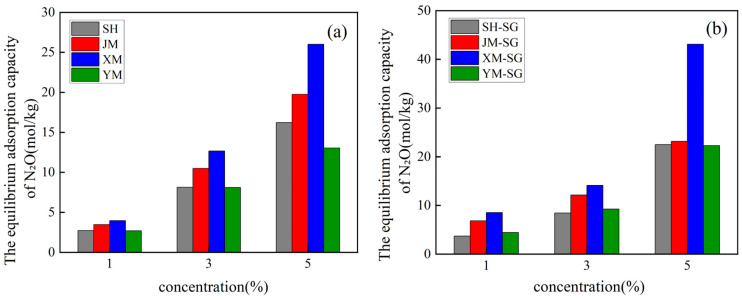
Equilibrium adsorption capacity of biochar for N_2_O gas before and after modification. (**a**) Before modification, (**b**) after modification.

**Figure 5:**
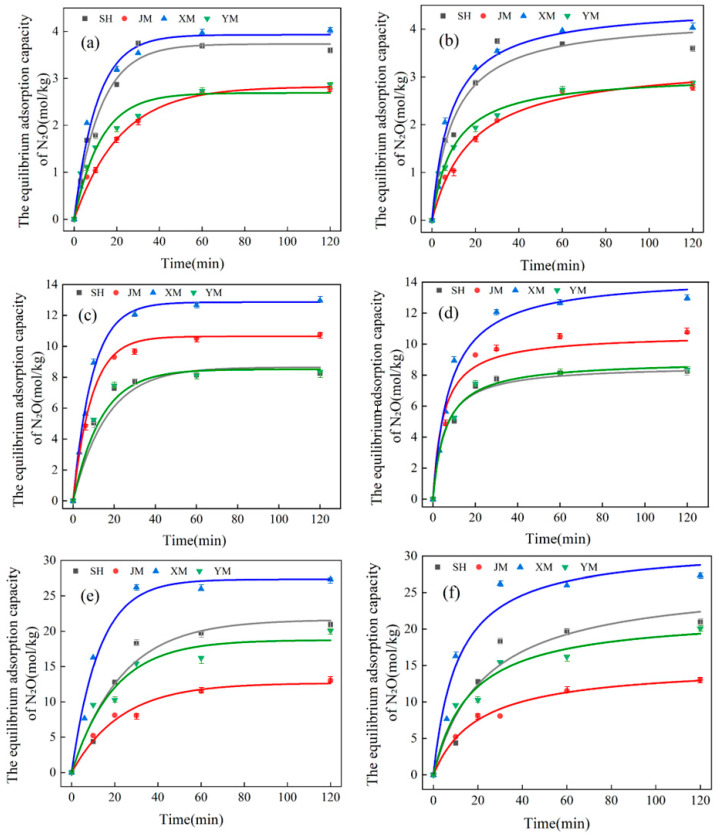
Quasi-first-order and quasi-second-order kinetic fittings of biochar before modification at concentrations of 1%, 3%, and 5%. (**a**) Quasi-first-order kinetic fitting before 1% modification, (**b**) quasi-second-order kinetic fitting before 1% modification, (**c**) quasi-first-order kinetic fitting before 3% modification, (**d**) quasi-second-order kinetic fitting before 3% modification, (**e**) quasi-first-order kinetic fitting before 5% modification, (**f**) quasi-second-order kinetic fitting before 5% modification.

**Figure 6:**
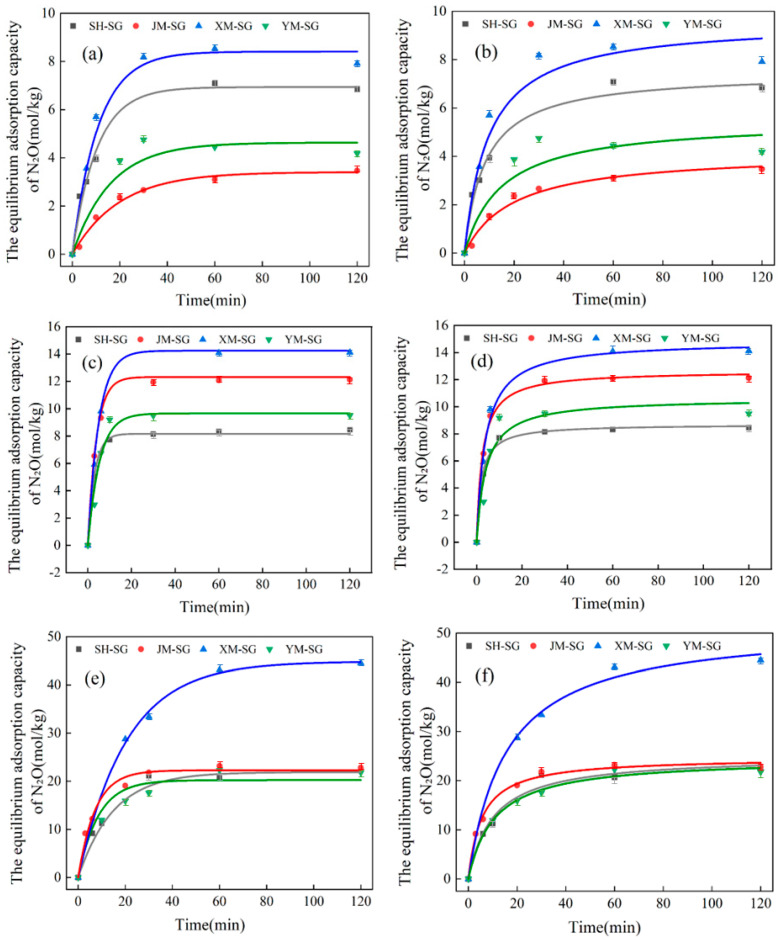
Quasi-first-order and quasi-second-order kinetic fittings of modified biochar at concentrations of 1%, 3%, and 5%. (**a**) Quasi-first-order kinetic fitting after 1% modification, (**b**) quasi-second-order kinetic fitting after 1% modification, (**c**) quasi-first-order kinetic fitting after 3% modification, (**d**) quasi-second-order kinetic fitting after 3% modification, (**e**) quasi-first-order kinetic fitting after 5% modification, (**f**) quasi-second-order kinetic fitting after 5% modification.

As shown in Figure 4, Figure 5 and Figure 6 and Table 6 and Table 7, all biochar materials reached adsorption equilibrium within a short timeframe at an initial N_2_O concentration of 1%, irrespective of modification status. Adsorption equilibrium was consistently achieved at 60 min across all tested initial concentrations. At an initial N_2_O concentration of 3%, the equilibrium adsorption capacities of SH, JM, XM, and YM were 8.141 mol/kg, 10.497 mol/kg, 12.670 mol/kg, and 8.109 mol/kg, respectively. For the acid-modified counterparts (SH-SG, JM-SG, XM-SG, YM-SG), the corresponding values were 8.462 mol/kg, 12.123 mol/kg, 14.094 mol/kg, and 9.252 mol/kg. At an initial N_2_O concentration of 5%, the equilibrium adsorption capacities of SH, JM, XM, and YM were 19.755 mol/kg, 13.038 mol/kg, 26.000 mol/kg, and 16.211 mol/kg, respectively. For SH-SG, JM-SG, XM-SG, and YM-SG, the corresponding values were 22.488 mol/kg, 23.158 mol/kg, 43.088 mol/kg, and 22.272 mol/kg. Across all tested concentrations, the equilibrium adsorption capacities followed consistent orders: XM > JM > SH > YM for unmodified biochars and XM-SG > JM-SG > SH-SG > YM-SG for acid-modified samples. All adsorption kinetics curves exhibited similar stage characteristics: a sharp initial increase in adsorption capacity, followed by a gradual deceleration, and ultimate saturation at equilibrium. Notably, the adsorption capacity of all biochars increased with rising initial N_2_O concentration, and acid-modified samples consistently showed higher adsorption capacities than their unmodified counterparts. Integrating the analyses of biochars before and after acid modification in Section 3.1, the optimized adsorption mechanism of acid-modified biochars for low-concentration N_2_O is proposed as follows: (1) Acid modification elevates the content of polar-oxygen-containing functional groups (e.g., -OH and -COOH) on the biochar surface, thereby enhancing adsorption affinity for polar N_2_O molecules [29,32]. (2) During the initial adsorption stage, the pore structure and high specific surface area of the biochar facilitate rapid N_2_O uptake, with gas molecules adsorbing onto the surface and filling the pores, leading to a sharp increase in adsorption capacity. However, as the reaction progresses, the reduction in available adsorption sites due to occupancy by N_2_O molecules results in a decline in both adsorption capacity and rate, eventually reaching equilibrium [33].

In light of the data presented in Table 6 and Table 7, both kinetic models adequately described the adsorption behavior of the biochars, both pre- and post-modification. However, comparative analysis of the R^2^ indicated that the pseudo-second-order kinetic model provided superior fitting accuracy. Pronounced disparities were noted in the adsorption rate constants and equilibrium adsorption capacities of SH, JM, XM, and YM across varying N_2_O concentrations. Both parameters exhibited an upward trend with increasing concentration, underscoring the positive effect of concentration on adsorption performance [34]. The adsorption rate constants (K_1_ and K_2_) served as indicators of adsorption kinetics, with higher values signifying more rapid adsorption and shorter equilibration times. XM consistently demonstrated elevated K_1_, K_2_, and R^2^ values across all concentrations, a trend mirrored in XM-SG, thereby validating its status as the most efficient adsorbent [35]. XM demonstrated remarkable adaptability: although its adsorption capacity increased more slowly at higher initial N_2_O concentrations, it achieved the highest cumulative equilibrium adsorption capacity among all materials, regardless of modification. Collectively, these findings highlight material composition, N_2_O concentration, and modification treatment as critical determinants of adsorption performance. The observed disparities in adsorption capacity are primarily attributable to variations in surface properties and chemical compositions among the materials.

### 3.3. Isothermal Adsorption Analysis of Biochars Before and After Acid Modification

**Figure 7:**
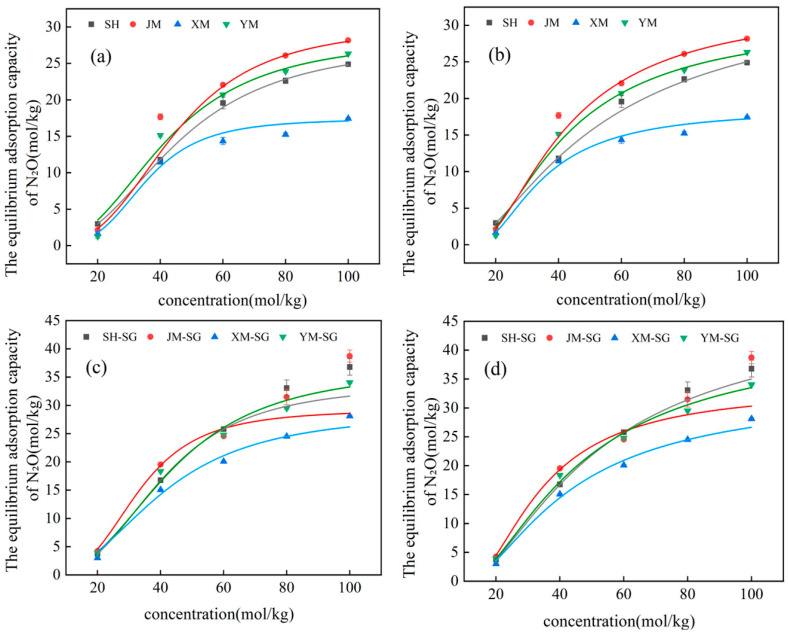
Isothermal adsorption fitting curves of biochar before and after modification. (**a**) Langmuir isothermal simulation before modification, (**b**) Freundlich isothermal simulation before modification, (**c**) Langmuir isothermal simulation after modification, (**d**) Freundlich isothermal simulation after modification.

Figure 7 further illustrates the N_2_O adsorption trends of different materials at varying concentrations. Notably, all materials—both unmodified and acid-modified—showed increasing adsorption capacities with rising N_2_O concentration. SH and XM consistently exhibited higher adsorption capacities across most conditions, highlighting their superior N_2_O capture capabilities. Comparative analysis of Figure 7 reveals a corresponding increase in equilibrium adsorption capacity for all materials with increasing initial concentration. At low concentrations, adsorption increased with N_2_O concentration, indicating that the process was dominated by concentration gradients due to abundant available adsorption sites. At medium concentrations, adsorption continued to rise but at a decelerated rate, likely resulting from partial occupation of adsorption sites. At high concentrations, adsorption capacity approached saturation. Significantly, acid-modified biochars showed notably higher adsorption capacities than unmodified counterparts, confirming that modification effectively enhanced both adsorption site density and capture efficiency.

Data in Table 8 and Table 9 reveal significant discrepancies in adsorption constants and equilibrium adsorption capacities of different biochars under Langmuir and Freundlich isothermal models. For instance, SH exhibited a Langmuir adsorption constant of 0.117 L/mol, equilibrium capacity of 27.356 mol/kg, and R^2^ of 0.998; under Freundlich model, its constants were 6.116 L/mol (adsorption constant) and 0.917 (adsorption index), with R^2^ = 0.909. JM showed Langmuir parameters of 0.126 L/mol (constant), 27.833 mol/kg (capacity), R^2^ = 0.968, and Freundlich parameters of 7.700 L/mol (constant), 0.840 (adsorption index), R^2^ = 0.824. Meanwhile, acid-modified SH-SG and XM-SG showed remarkably enhanced Langmuir equilibrium capacities: 33.809 mol/kg for SH-SG and 36.604 mol/kg for XM-SG. Integrating the characterization results and discussions in Section 3.1, acid modification elevated oxygen content in SH-SG, XM-SG, and YM-SG, with SH-SG showing higher levels. Increased oxygen-containing functional groups (e.g., hydroxyl, carboxyl) correlated with enhanced N_2_O adsorption across all materials. The adsorption capacity order (XM-SG > SH-SG > JM-SG > YM-SG) mirrored elemental analysis, validating oxygen functionalization’s critical role in N_2_O capture, likely because increased oxygen content manifested as more polar functional groups (e.g., hydroxyl/carboxyl), thereby enhancing adsorption of polar N_2_O molecules [36]. For all four biochars, Langmuir model fitting outperformed Freundlich model fitting. The Freundlich parameter 1/*n* reflects adsorption performance; lower values of 1/*n* signify enhance adsorption. Specifically, when the value of 1/*n* falls between 0 and 1 (0 < 1/*n* < 1), it generally indicates favorable adsorption conditions, whereas values greater than 1 suggest poor adsorption performance [37]. In this study, all fitted 1/*n* values were within the range of 0 to 1, thereby confirming the excellent adsorption performance of both unmodified and modified biochars.

Comprehensive analysis of Table 8 and Table 9 and Figure 7 shows acid modification significantly enhanced biochars’ N_2_O adsorption capacity, attributed to increased specific surface area and active sites. Adsorption characteristics varied among materials: SH-SG and XM-SG showed the highest capacities, while modified JM-SG and YM-SG had moderate improvements. Both Langmuir and Freundlich models fitted well, indicating mixed monolayer adsorption and surface heterogeneity. Isotherms confirmed modified samples had significantly higher capacities, especially at high N_2_O concentrations, validating modification effectiveness.

## 4. Conclusions

This study prepared biochar from four agricultural wastes: pecan shells (SHs), poplar sawdust (JM), wheat straw (XM), and corn straw (YM). We systematically investigated the effects of acid modification biochar on low-concentration N_2_O adsorption performance, yielding three key conclusions: (1) Acid modification significantly altered the elemental composition, pore structure, and surface functional groups of biochars, manifested as increased oxygen content, decreased carbon content, reduced graphitization, enhanced specific surface area/micropore volume, and enriched surface functional groups (e.g., -OH, -COOH). These physical structural optimizations provided more adsorption sites for gas molecules, thereby improving the low-concentration N_2_O adsorption capacity of modified biochars. (2) Modified biochars exhibited remarkable performance enhancement in N_2_O adsorption, featuring faster kinetic rates and higher equilibrium adsorption capacities. Both Langmuir and Freundlich isothermal models showed good fitting degrees, indicating that the adsorption behavior involved both monolayer adsorption characteristics and surface heterogeneity. (3) Introducing more oxygen functional groups (-OH, -COOH) and optimizing pore structures significantly improved biochars’ adsorption performance. -OH and -COOH played critical roles in the adsorption process by enabling chemical reactions with low-concentration N_2_O molecules, while the optimized pore structures provided additional adsorption sites to enhance efficiency. The modified biochars not only effectively reduce greenhouse gas emissions but also demonstrate high economic feasibility and environmental friendliness.

## Figures and Tables

**Table 1:** Biochar preparation conditions.

Material	Preparation Temperature (°C)	Heating Rate (°C/min)	Holding Time (h)
pecan shell biochar (SH) [19]	600	10	1
poplar sawdust biochar (JM) [20]	700	5	2
wheat straw biochar (XM) [21]	550	5	4
corn straw biochar (YM) [22]	800	5	2

**Table 2:** Elemental composition of biochar before modification.

Material	C/%	O/%	H/%	N/%	Ash/%	H/C	O/C	(O + N)/C
SH	85.72	8.85	2.87	0.70	1.88	0.033	0.103	0.111
JM	68.04	12.34	2.10	0.92	16.61	0.029	0.181	0.205
XM	37.84	6.86	1.65	2.20	52.30	0.037	0.253	0.282
YM	42.92	7.38	1.26	3.19	47.34	0.028	0.301	0.352

**Table 3:** Elemental composition of biochar after acid modification.

Material	C/%	O/%	H/%	N/%	Ash/%	H/C	O/C	(O + N)/C
SH-SG	83.11	10.91	2.47	0.30	1.01	0.030	0.131	0.135
JM-SG	81.65	10.88	1.84	0.23	5.41	0.023	0.133	0.136
XM-SG	38.17	8.17	1.84	0.87	50.96	0.048	0.214	0.237
YM-SG	44.87	7.99	1.29	0.66	45.20	0.029	0.178	0.193

**Table 4:** Specific surface area and pore size structure of biochar before modification.

Material	Specific Surface Area m^2^/g	Total Pore Volume cm^3^/g	Micropore Volume cm^3^/g	Aperture nm
SH	3.3833	0.006687	0.000489	32.0450
JM	190.3343	0.103057	0.059245	4.6607
XM	14.1885	0.031243	0.001688	8.3111
YM	134.3059	0.087114	0.041573	7.3314

**Table 5:** Specific surface area and pore size structure of biochar after acid modification.

Material	Specific Surface Area m^2^/g	Total Pore Volume cm^3^/g	Micropore Volume cm^3^/g	Aperture nm
SH-SG	472.4921	0.196466	0.160959	3.2040
JM-SG	312.4882	0.157832	0.100254	3.9573
XM-SG	84.3999	0.068609	0.024710	8.1966
YM-SG	203.2276	0.113235	0.065553	5.9984

**Table 6:** Kinetic analysis of N_2_O gas adsorption by biochar before modification.

Material	N_2_O Concentration	Pseudo-First-Order Kinetics			Pseudo-Second-Order Kinetics		
		Adsorption Constant *k*_1_ L/min	Equilibrium Adsorption Capacity *q_e_* mol/kg	R^2^	Adsorption Constant *k*_2_ kg/mol/min	Equilibrium Adsorption Capacity *q_e_* mol/kg	R^2^
SH	1%	0.081	3.731	0.953	0.023	4.256	0.976
	3%	0.061	8.648	0.867	0.022	8.647	0.985
	5%	0.043	21.644	0.914	0.002	26.687	0.943
JM	1%	0.046	2.824	0.974	0.016	3.330	0.988
	3%	0.110	10.640	0.877	0.019	10.640	0.972
	5%	0.044	12.664	0.969	0.003	15.102	0.984
XM	1%	0.093	3.928	0.978	0.025	4.492	0.981
	3%	0.106	12.855	0.980	0.009	14.421	0.994
	5%	0.079	27.338	0.949	0.003	31.422	0.977
YM	1%	0.080	2.689	0.944	0.033	3.060	0.980
	3%	0.073	8.508	0.919	0.019	8.942	0.987
	5%	0.051	18.758	0.938	0.003	22.003	0.960

**Table 7:** Kinetic analysis of N_2_O gas adsorption by modified biochar.

Material	N_2_O Concentration	Pseudo-First-Order Kinetics			Pseudo-Second-Order Kinetics		
		Adsorption Constant *k*_1_ L/min	Equilibrium Adsorption Capacity *q_e_* mol/kg	R^2^	Adsorption Constant *k*_2_ kg/mol/min	Equilibrium Adsorption Capacity *q_e_* mol/kg	R^2^
SH-SG	1%	0.097	6.937	0.979	0.016	4.743	0.979
	3%	0.324	8.166	0.994	0.059	8.705	0.994
	5%	0.067	21.883	0.933	0.004	25.037	0.974
JM-SG	1%	0.050	3.410	0.976	0.015	4.000	0.985
	3%	0.257	12.319	0.991	0.032	12.643	0.995
	5%	0.138	22.271	0.981	0.007	24.722	0.993
XM-SG	1%	0.106	8.320	0.961	0.016	9.174	0.990
	3%	0.200	14.242	0.991	0.018	14.834	0.994
	5%	0.049	44.846	0.994	0.001	51.612	0.998
YM-SG	1%	0.123	4.434	0.966	0.167	4.465	0.975
	3%	0.188	9.656	0.910	0.024	10.614	0.967
	5%	0.068	21.794	0.985	0.004	24.595	0.989

**Table 8:** Adsorption capacity of biochar for N_2_O gas before modification.

Material	Langmuir			Freundlich		
	Adsorption Constant *k_l_*	Equilibrium Adsorption Capacity *q_e_* mol/kg	R^2^	Adsorption Constant *k_F_*	Adsorption Index 1/*n*	R^2^
SH	0.117	27.356	0.998	6.116	0.917	0.909
JM	0.126	27.833	0.968	7.700	0.840	0.824
XM	0.134	16.578	0.966	5.254	0.784	0.791
YM	0.097	26.071	0.977	5.572	0.899	0.837

**Table 9:** Adsorption capacity of modified biochar for N_2_O gas at room temperature.

Material	Langmuir			Freundlich		
	Adsorption Constant *k_l_*	Equilibrium Adsorption Capacity *q_e_* mol/kg	R^2^	Adsorption Constant *k_F_*	Adsorption Index 1/*n*	R^2^
SH-SG	0.007	33.809	0.999	6.793	0.908	0.944
JM-SG	0.022	29.233	0.954	7.889	0.953	0.947
XM-SG	0.055	36.604	0.970	7.884	0.930	0.927
YM-SG	0.062	28.820	0.991	6.696	0.879	0.927

## Data Availability

The original contributions presented in this study are included in the article. Further inquiries can be directed to the corresponding author.
